# The immune system as a regulator of normal physiology

**DOI:** 10.3389/fimmu.2026.1867359

**Published:** 2026-07-06

**Authors:** John V. Forrester, Lucia Kuffova, Andrew D. Dick

**Affiliations:** 1 University of Aberdeen, School of Medicine, Medical Sciences and Nutrition, Institute of Medical Sciences, Aberdeen, United Kingdom; 2 INSiGHT Comprehensive Eye Care, Hamilton, Bermuda; 3 Academic Unit of Ophthalmology, Translational Health Sciences, University of Bristol, Bristol, United Kingdom; 4 NIHR Biomedical Research Centre of Ophthalmology, Moorfields Eye Hospital, London, United Kingdom; 5 University College London (UCL) Institute of Ophthalmology, London, United Kingdom; 6 School of Biochemistry and Biomedical Sciences, University of Bristol, Bristol, United Kingdom

**Keywords:** immunity, infection, latent, microbiome, tolerance

## Abstract

Concepts of how the immune system functions have evolved during the last half century. From widespread acceptance of Self-Nonself Discrimination to explain adaptive immunity, to increasing understanding of receptor-mediated activation of innate immunity through the Danger Model, understanding of the role of the immune response continues to grow with ever broader models such as the Damage Response Framework and the Discontinuity Model. The realisation that the majority of foreign antigens, such as those comprising the microbiome, are tolerated by the immune system, allows a re-appraisal of immune tolerance and how it relates to these conceptual shifts. Disease induced by both autoantigens and foreign antigens occurs most readily when the abundantly redundant immune system is defective, for genetic or other reasons, in one or more critical component. When fully competent, the primary role of the immune system is to “physiologically manage” both foreign and self-antigens by quietly engaging in activities such as waste disposal, autophagy, removal of apoptotic cell debris, tissue repair and metabolism. This is evidenced for foreign antigens by the limited incidence of clinical disease in pandemics despite widespread exposure of the population to the infectious agent: instead, many asymptomatic infected individuals harbour latent infections controlled by a healthy immune system, and are mainly at risk of developing overt disease when immune competency is impaired. In the case of autoantigens, by definition, exposure is 100% yet disease incidence is minimal and similarly requires a failure of immune competence, initiated by the same aberrant response to a coincident foreign antigen.

## Introduction

Immunology developed as a separate discipline in the wake of 19th century germ theory (1, 2) with the discovery of innate (3) and adaptive (4) immune responses to microbes. Recognition of microbes by innate immune cells (macrophages and other myeloid cells) was considered non-specific while recognition of antigens, particularly protein antigens, by newly discovered T and B cells (5) was shown to have exquisite specificity through the T and B cell receptor (TCR, BCR) repertoires. However not all antigens generated immune responses, particularly host (self) antigens, and the concept of self-nonself discrimination (SNSD) introduced by Burnet (6) grew as a central plank of how the immune system functioned [reviewed in (7)]. Non-responsiveness by the immune system to host antigens was described as immunological tolerance and loss of tolerance, i.e. immune responsiveness to self-antigens, was described as autoimmunity, the associated pathology being autoimmune disease. Most of the initial response to challenge was considered the responsibility of the innate immune system (8, 9) which constituted the main defence against pathogens, together with “non-immunological” components determining tissue composition (yolk-sac derived cells, stromal fibroblasts etc.) and architecture (tissue barriers to pathogen entry). In contrast, the adaptive immune response constituted a memory (recall) response with greater efficiency when re-challenged with the recall antigen.

In time, innate immune responses by myeloid cells to pathogens were shown also to be receptor-mediated (10, 11) through class-based, microbe-restricted ligands. Receptor organisation was much broader for innate immune cells which recognised 5 main categories of microbe (viruses, bacteria, archaea, fungi and protists) mainly through a wider range of shared microbial components including proteins, ssRNA, sugars/lectins, LPS and lipids. However, with realisation that host “commensal” organisms could form a discrete mixed population defined by their location (a microbiome) (12) and that their genetic composition (metagenome) could be described (13) it became clear that the immune system’s interaction with microbes rarely generated a response. Koch himself recognised this fact. Indeed, beyond the host microbiome, only 1,400 of planet earth’s estimated one trillion species of microbe (14) are considered human pathogens (15). This has considerable implications for understanding the pathogenesis of autoimmune disease (16) and possibly even the primary function of the immune system which may simply be one of maintaining physiological homeostasis whilst tissues in return variously exert control over the immune response.

This perspective considers the various mechanism by which the immune system may operate.

## How does the Immune system work?

### Self-nonself discrimination

SNSD as a concept was helpful in elucidating the machinery of T and B cell responses, grounded on the vast potential repertoire of T and B cell receptors for recognising antigens. SNSD was based on the assumption that specific receptors could react positively to self-antigens by activating signalling pathways leading to measurable outcomes such as deletion or anergy. As such, self-antigens were considered “tolerated” by the immune system (immune tolerance). However, scrutinous testing of SNSD revealed that host (self) antigens could induce a classical immune response (IR) if delivered in the appropriate context. Importantly, there was no fundamental mechanistic difference in the IR to self vs non-self-antigens [reviewed in (4)]. Specifically, it was stated that, if self-antigens induced an IR, “the effector mechanisms underlying autoimmune disease are the same as those responsible for immunity against invading microorganisms “. This has not changed in the ensuing 20+ yrs since first articulated and it was accepted that self-antigens for unknown reasons did not induce a pathological IR in the healthy individual.

Viewed from an opposing perspective, it is also true that most nonself antigens as well as self-antigens fail to generate pathological IRs. In fact, the explosion of information concerning the microbiome shows that tolerance to the trillions of antigens in the host microbiome is the norm and that immunogenicity to foreign antigens is unusual if not rare (this applies also at the molecular level - protein antigens may be constructed of many peptide epitopes but only a few are immunogenic). Explanations for tolerance to the “commensal” microbiome may include intrinsic features of certain microbes but are mainly considered in terms of factors which prevent IRs like secreted products (short chain fatty acids) or physical barriers (dense surface mucus layers at mucosal surfaces) or immune regulatory cells (adaptive and innate immune cells). Indeed, a richly diverse microbiome can have beneficial effects by reducing the risk of Type 2 IRs (17). These data have necessitated a re-think of SNSD (16), one interpretation being that the immunogenicity of an antigen is less dependent on its source (self vs non-self), that it may vary over time, and that it is context dependent.

SNSD as originally formulated was considered only in reference to adaptive immunity. With greater understanding of the complexity of the immunogenic response (such as the need for co-stimulation), it was realised that tolerogenic adaptive responses not only involved T and B cell recognition of antigen but also involved different signalling pathways (such as regulatory pathways) (18) and diverse T (18) and B (19) cell responses. Indeed, autoreactivity has been estimated to occur in 20-50% of VDJ recombination events (20). Soon, it was realised that autoimmune responses in the form of autoantibodies and autoreactive T cells were part of immunological homeostasis and were probably transient “physiological” responses leading to one of four potential outcomes (20). In fact, as Goodnow et al. state (20), “the considerable scope for tolerating self-reactive receptors” strongly suggests a physiological response. Consequently, the immune system’s role in providing protection against pathogens may be a component of a more fundamental house-keeping role in, for example, clearing physiological waste and ensuring optimal cellular and metabolic function. Furthermore, in the case of T cells, the SNSD argument also breaks down when receptor specificity is seen to be degenerate (21). Indeed, recent studies of the immunopeptidome, for example, have shown extensive “polyspecificity” and immunogenicity of selected environmental antigens for the TCR in patients with psoriasis, a condition considered autoimmune in pathogenesis (22). Perhaps it is in the realm of T regulatory (Treg) cell induction that the SNSD concept retains relevance: currently, thymic (t)Tregs are selected for specificity to self-antigen under control of the *Aire* gene, whilst peripheral (p)Tregs are converted from thymic emigrants in response to commensal antigen (23). However, this is not SNSD as originally conceived. Furthermore, Tregs are not only induced by “harmless” commensals but play a major role in control of the pathogenesis of infection (24, 25).

### Danger theory

The discovery of pathogen recognition receptors (PRRs) which reacted to different categories of microbe, initially in the fruit fly (26) and then in humans [reviewed in (10)], led to a new model of how IRs were initiated. Cells involved in antigen presentation (APCs) to T cells were found to be selectively activated by distinct classes of pathogen-associated molecular patterns (PAMPs) which bound to PRRs, generating sets of proinflammatory molecules (cytokines) and enabling antigen-specific T cell activation. PRRs were initially considered to support SNSD through upregulation of co-stimulatory molecules required for induction of adaptive immunity (27). While they were abundant on APC’s such as dendritic cells and macrophages, they were also present on non-immune cells, particularly epithelial cells lining surface barriers. Their activation, leading to the generation of cytokines, chemokines and other reactive molecules, induced entry-site recruitment and activation of APCs and induction of both innate and adaptive immunity. Matzinger enlarged on this concept of how the immune system, both innate and adaptive, was activated and suggested that “danger” signals (danger-associated molecular patterns, DAMPS) were what initiated the IR (28). This imprecise notion was modified to “damage” signals which allowed easier measurement whilst retaining the concept of DAMPs. More specifically, DAMPs have been identified as “alarmins”, proteins released by cells and tissues under stress or damaged by pathogens with the ability to activate immune cells, particularly those involved in Type 2 immunity (29, 30). Several specific alarmins have been described in association with pathogens (e.g., defensins, HMGB-1, IL33) but are also released in “sterile” damage not directly involving microbes. The same alarmins are also big players in the pathogenesis of typical autoimmune diseases, both at the innate immune antigen presentation level (29) as well as during TCR engagement where antigen specificity can be effectively diluted when the TCR responds to multiple self and foreign antigens (22).

The DAMP/PRR model of immunity restored the significance of innate immunity in the response of the host to challenge, especially immunological challenge. While adaptive immunity is essentially memory-dependent and equips the host for future rapid and heightened host responses to the initial pathogen, innate immunity deals with the immediate survival response and mobilises a wide array of defence armoury to clear the pathogen including cell migration, phagocytosis, neutrophil-mediated NETosis, free radical production and more, with a quasi-selective nature given the distribution of TLRs. Its broader specificity allows it to activate the signalling pathways necessary to deal with categories or sets of microbes and thus clear the pathogen more efficiently. Innate immunity even has a degree of memory, termed trained immunity, which is retained for weeks to months (31), thus allowing it to cope with locally proliferating microbes which have escaped initial clearance. However, some microbes may still evade the immune system and persist within cells as latent infections, kept under control by the adaptive immune system (32, 33). Matzinger has emphasised the role of the tissue in this respect, particularly sites of immune privilege (IP) which provide preferential niches for latent infection (34).

In her obituary for Charles Janeway, Matzinger stated that Janeway’s concept of the immune system was that its “ default state is *off* and it could be turned on by bacteria” (35); we suggest its default state is more likely “tick-over” whilst it deals with housekeeping jobs such as clearance of waste and apoptotic cell debris.

### Damage response framework of immunity

Casadevall and colleagues have an alternative take on the host/microbe interaction (36) emphasising the prominent role of the host response. They focus on pathogenicity, defining a pathogenic microbe as one that is capable of causing damage to the host; they describe this as the damage response framework (DRF) (36). This permits them to consider microbes, particularly those causing opportunistic infections, and latent infections, in terms of the host response. Autoimmune, and especially auto-inflammatory conditions are considered to occur in individuals who have a genetic defect in their innate immune system and are induced by microbes which are pathogenic only under special circumstance. For instance, the prioritization of genetic variants in autoimmune disease and its potential skewing of naive T cells to become effector T cells triggered by viral infection has recently been reported (37, 38). Autoinflammatory conditions are often the result of activity by microbes which normally colonise the skin or mucosa as commensal microbiome but do not normally cause damage. A clear example is cystic fibrosis where obstructive lung infection by *P.aeruginosa* causes fatality in patients with mutations in the CFTR gene [encoding cystic fibrosis transmembrane regulator (CFTR)] (39) but are normally non-pathogenic in healthy individuals. Autoimmune conditions and the adaptive immune response might be entrained through mechanisms such as molecular mimicry between autoantigenic and microbial epitopes but pathology is dependent on the host response which might range from nil (latent infection) to extreme (fatal infection). The focus of the DRF is predominantly on damage-causing microbes rather than inclusive of all types of perturbation and the DRF classification of pathogens is dependent on unspecified “strong” and “weak” host IRs. How the DRF relates to the IR is therefore unclear but in terms of infectious disease, Casadevall and Pirofski consider that infectious disease (ID) occurs when the immune response is either insufficient to control microbial activity or when it controls microbial cytotoxicity but causes excessive host damage. In either case, this interpretation of immunity subsumes the question of autoimmunity into host damage and is of considerable practical value to the physician attempting to treat disease. For instance, presumed autoimmune, non-infectious uveitis probably centres directly or indirectly on an infectious etiology (40), but its management is one of minimising damage by control of inflammation.

### Host theory of immunity

Casanova also considers that the role of the host in the IR to infectious agents is under-represented (41). The basis for this concept enlarges on Dubos’ observation that for any infectious disease, only a limited number of individuals are clinically affected and few severely so, even though infection may be widespread. Like Casadevall, Casanova relates this to host susceptibility, recently demonstrated in the COVID-19 pandemic where severely affected individuals were shown to have inborn errors of immunity, such as type 1 IFN immunity involving IFNAR1 or IRF7 deficiency (42, 43). Similar observations were made on susceptibility to a range of infections including mycobacterial infection (MSMD) involving a rare *TYK2* variant with deficient IL23-dependent IFNγ production (44, 45), herpes simplex encephalitis and aberrant TLR3 signalling through RIPK3 (46, 47), and the multiple immune defects in APECED syndrome caused by deficiency in the tolerance regulatory gene *Aire* (48). Casanova views the IR as essentially host driven and that it only truly serves its homeostatic function when the myriad genes regulating both innate and adaptive immunity are optimally functional. Indeed, due to considerable genetic and functional redundancy, the IR maintains a high level of consistency and reproducibility (49, 50). This redundancy allows healthy individuals to manifest considerable variability in IRs to cope with acute changes as well as circadian alterations. Casanova does not accept that “immunocompetent” individuals can succumb to death by infection. This polarised view may have a lot to support it, particularly if the role of the immune system is one of maintaining homeostasis, and only fails when its physiological functions are defective. This would be equivalent to Casadevall’s “weak” immune response and the link to variant genetic risk (see above).

The immune system can thus be viewed as a waste removal, tolerogenic system with the capacity to deal silently with self and microbial products, and can thus be set amongst other parallel homeostatic systems including metabolism, digestion, the CNS, renal function, etc. Only in the absence of a bespoke, fully functional system (defective immunity) or when the conditions/context changes (e.g., trauma) will damage occur and disease ensue.

### Discontinuity theory of immunity

According to Casanova, Dubos - a pioneer of antibiotics in the twentieth century - strongly held the view that the immune system was guided by “changes in circumstances” particularly ecological circumstances (41). In recent years, Pradeu has further promoted this concept. On the basis that all receptors, including photoreceptors, follow Weber’s Law of receptor adaptation to incremental change in the strength of stimulation (51), Pradeu has proposed a “Discontinuity Theory” of immunity in which the immune system is not restricted in its responses to foreign antigen or to danger, but responds to change in the timing of exposure to a given immune stimulus whether it be danger, pathogen or metabolic change (52–54). Continuous (co-)stimulation (“weak” or chronic) elicits a tolerogenic response whilst discontinuous stimulation (“sudden” interaction with pathogen, tumour antigen, metabolic change etc.) elicits an effector (immunogenic) response involving innate immunity initially, followed by an adaptive response. Sudden discontinuous stimuli include recognition of PAMPs on microbes, DAMPs in tissue damage, as well as rapid physiological changes in the cell. In contrast, continuous stimuli include host self-antigens, the microbiome, and a more recent concept of disease tolerance, a defence mechanism that spares the tissue whilst permitting pathogen persistence (55, 56). This correlation of IR with speed of change in exposure to an immunological stimulus permits an explanation of why the same stimulus can induce both tolerance and immunity. In one sense the adaptive memory response fits with this concept in that it generates a more rapid specific response to re-challenge. Pradeu cites a range of supportive experimental studies and identifies a set of “hallmarks” of immunity (53, 54).

## The immune system as a regulator of normal physiology

As concepts of immunity and its molecular and cellular actors have expanded from the SNSD paradigm of adaptive immunity, to the Danger Theory of innate immunity, to an appreciation of immunity as monitor of change or perturbation in the context of the host, it can be inferred that the primary function of the immune system is not simply to protect the host from pathogen challenge but to maintain normal physiological processes, as proposed by Pradeu. The main actors in this are regulatory cells, particularly T regulatory cells (Tregs) (57–60), which are not only the lynchpin of immune tolerance but have several other roles such as promoting metabolic stability (61), promoting neurogenesis and brain physiology (62), and as overseers of barrier integrity (63). In the steady state resident myeloid cells also populate the tissues extensively and amplify “house-keeping” functions of cells generally, both through external (clearance of dead and dying cells, synaptic trimming, trogocytosis) and internal (autophagy) mechanisms. Gut commensals play an important part in this by acting on local DC to release Type1 IFN which activates production of IL10 and IL27, thereby generating Tregs (64, 65). The wider involvement of immune cells in tissue repair and surveillance [Tregs (66, 67), tissue-resident macrophages (68, 69)] occurs in the context of “tonic” levels of inflammation [para-inflammation (70, 71)] whilst promoting immunological tolerance both of self and non-self-antigens (e.g. commensals) via mechanisms such as linked suppression (72).

Appreciation of the physiological functions of the immune system does not reduce its protective function in the face of pathogen. When called upon, the immune system responds rapidly and effectively to challenge and is especially equipped for this role via the innate immune response. The adaptive immune response is mainly built around readiness for future attacks by pathogens it has met previously. This it can do with fine-tuned sensitivity involving sophisticated molecular mechanisms (immunodominance, TCR competition, receptor editing, MHC:peptide stability etc.) leaving a legacy of surveillant tissue resident memory cells. However, the default response of the immune system both to self and foreign antigens is determined by the host and is one of quiescence.

## Autoimmunity as a by-product of infection

The above discussion has a number of implications. The first is that immunity to Self is not in essence different from immunity to Nonself. The same processes of innate and adaptive immunity are entrained when the host maintains homeostasis (tolerance) to self or foreign antigens. Recognition of either in a changed context can induce the host to mount a response ultimately aimed at restoring homeostasis and as Eberl and Pradeu suggest (52), damage may occur if the host cannot adapt quickly enough to the change. This may be because the adaptation is either insufficient or exaggerated.

Maintaining homeostasis is achieved by providing physical, cellular and molecular barriers and a rapidly-responding innate immune system designed to clear the pathogen quietly and efficiently. This is rarely 100% effective, but provided the initial challenge is not lethal, residual microbes may persist in some form as latent infections (33, 73). The immune system is intimately involved in this, particularly so during viral infections such as EBV and HSV but is also true of other classes of pathogen (73). Most recently, RNA viruses have also been similarly inculpated (74). The ongoing AIDS epidemic and the increased use of immunosuppressive therapies have revealed the extent and range of silent/hidden infection which is present in the immunocompetent population. Interestingly, some of these hidden latent infections are now implicated as a pathogenetic fulcrum of so-called autoimmune disease such as EBV and multiple sclerosis (75). In fact, when infection spreads through a population, most infected individuals are asymptomatic but often harbour latent infection which may resurface many years later (e.g. herpetic infections, mycobacterial infection, fungal infections) (33, 73). Most latent infections do not resurface but can do so as overt infections when the adaptive immune defence mechanisms are aging or defective. This also applies to some age-related, degenerative and chronic inflammatory diseases where links to a previous infection are increasingly recognised such as rheumatoid arthritis (76–78), Alzheimer’s disease (79), macular degeneration (80, 81) and many more.

## The immune system today

From the initial descriptions of non-specific innate immunity to the highly sophisticated antigen receptor-based adaptive immunity, it is recognised that the innate immune system provides the lion’s share of initial defence whilst the adaptive immune system provides memory. However, the perception of the immune system simply as a pathogen defence mechanism has transformed into being viewed much more broadly as a guardian of homeostasis. This evolved from an understanding of immune tolerance and SNSD to a position where “immune cells” such as T regulatory cells are now recognised to contribute towards regulation of metabolism (61, 82) and promotion of tissue repair (57, 61, 67) as part of the DRF. In this context, a two way interaction between immune and non-immune cells such as fibroblasts (83) and epithelial cells (84), may have a decisive role to play, not only in terms of barrier function but in shaping molecular signalling events required for homeostasis, for instance in the eye and the brain (85, 86). While its role in immune defence remains a core function and is dependent on other factors such as context, microenvironment and pathogen virulence, the immune system’s interaction with the world of microbes, especially commensals, may even be one of co-operation in the pursuit and maintenance of homeostasis. This is a delicate balance requiring adaptation and evolution by both host and pathogen respectively, and is reflected in variable progression of pandemics and their mode of transmission. Disruption of homeostasis is readily detected if the change is sudden (Discontinuity Theory) but in the resting state the immune system’s main physiological function is a “housekeeping” one of trimming the tissues of dead and dying cells through mechanisms such as autophagy and phagocytosis without activating inflammatory mechanisms and repairing tissues where needed. Vaccination against potential pathogens is an interventional manipulation of the immune system which pre-empts disruption and promotes homeostasis, and can be achieved by inducing pathogen-specific memory via adaptive immunity as well as innate immunity (31, 69).

## Conclusion

The role of the immune system is to maintain homeostasis of the host by managing non-microbial antigens (including self-antigens) as well as microbial antigens without inducing host damage in the process. Symbiosis/commensalism is one *modus vivendi* by which this is achieved, as is post-infectious microbial latency. In this respect the immune system is similar to other homeostatic regulators. Latent infections and carrier status are therefore common. However, when (less commonly) host survival is threatened, the innate immune system has the capacity to respond rapidly to clear the pathogen which places the host at risk of incurring damage particularly when the immunological machinery is defective. Meanwhile, the adaptive immune system stores memory in preparedness for future challenge. Regulatory cells (lymphoid and myeloid) are central to both physiological homeostasis and recovery from injury, and in the case of Tregs operate with a degree of tissue specificity/adaptation (62, 87).

## Data Availability

The original contributions presented in the study are included in the article/supplementary material. Further inquiries can be directed to the corresponding author.
